# Neural Mechanisms of the Impact of Rotated Terrain Symbols on Spatial Representation in Orienteers: Evidence from Eye-Tracking and Whole-Brain fNIRS Synchronization

**DOI:** 10.3390/bs15101314

**Published:** 2025-09-25

**Authors:** Shijia Ou, Tianyu Liu, Yang Liu

**Affiliations:** 1 School of Physical Education, North East Normal University, Changchun 130024, China; oushijia125@nenu.edu.cn; 2 School of Physical Education, Shaanxi Normal University, Xi’an 710119, China

**Keywords:** orienteering, terrain symbols, spatial representation, eye-tracking, functional near-infrared spectroscopy (fNIRS)

## Abstract

Spatial representation is a core element of spatial cognition in orienteering, but the visual-spatial neural modulation mechanisms underlying spatial representations with differently oriented maps have not yet been systematically elucidated. This study recruited 67 orienteering athletes as participants and employed a single-factor (map orientation: normal vs. rotated) between-subjects experimental design. Eye-tracking and functional near-infrared spectroscopy (fNIRS) techniques were used simultaneously to collect behavioral, eye movement, and brain activity data, investigating the effects of map orientation on visual attention and brain activity characteristics during terrain symbol representation processing in orienteering athletes. The results revealed that compared to the normal orientation, the rotated orientation led to significantly decreased task accuracy, significantly prolonged reaction times, and significantly increased saccade amplitude and pupil diameter. Brain activation analysis showed that the rotated orientation elicited significantly higher activation levels in the right dorsolateral prefrontal cortex (R-DLPFC), bilateral parietal lobe cortex (L-PL, R-PL), right temporal lobe (R-TL), and visual cortex (VC) compared to the normal orientation, along with enhanced functional connectivity. Correlation analysis revealed that under normal map orientation, accuracy was positively correlated with both saccade amplitude and pupil diameter; accuracy was positively correlated with activation in the R-DLPFC; saccade amplitude was positively correlated with activation in the R-DLPFC and R-PL; and pupil diameter was positively correlated with activation in the R-DLPFC. Under rotated map orientation, accuracy was positively correlated with saccade amplitude and pupil diameter, and pupil diameter was positively correlated with activation in both the L-PL and R-PL. The results indicate that map orientation significantly influences the visual search patterns and neural activity characteristics of orienteering athletes, impacting task performance through the coupling mode of visual-neural activity.

## 1. Introduction

The global surge in outdoor adventure activities has heightened concerns regarding safety in mountain navigation. The 2024 Annual Report on Outdoor Adventure Accidents in China reveals that 81.8% of the 335 reported accidents occurred in mountainous environments, with becoming lost and stranded identified as the primary risk factors. This underscores a widespread deficiency among those in distress: the lack of map symbol-terrain spatial transformation ability. Specifically, individuals struggle to construct accurate three-dimensional (3D) mental representations of terrain based on terrain symbols such as contour lines and saddles, leading to misjudgments of location and failures in route planning (Clark et al., 2008). This phenomenon highlights a core discrepancy in outdoor safety: the theoretical navigational value of maps is disconnected from users’ practical ability to construct spatial representations based on terrain symbols. Terrain symbol representation refers to an individual’s capacity to interpret abstract symbols on a map and thereby construct, manipulate, and utilize spatial information about the corresponding 3D topographic structure in their mind (Atit et al., 2016). This ability constitutes the core cognitive foundation for safe navigation outdoors, particularly in mountainous environments. Consequently, investigating how individuals efficiently and accurately form representations of mountainous terrain to achieve successful mountain navigation, and elucidating the underlying cognitive and neural mechanisms, represents a critical research direction within the field of spatial cognitive science.

Orienteering, an outdoor sport that heavily relies on map navigation to locate checkpoints rapidly within complex mountainous terrain (Waddington & Heisz, 2024), offers a highly ecologically valid research model for investigating spatial representation abilities. During mountain navigation practice, orienteering athletes must perform dynamic spatial transformations from two-dimensional (2D) map symbols (such as contour lines and saddles) to three-dimensional (3D) topographic structures in real-time. They must also continuously adjust map orientation and travel direction to optimize route decision-making. Research indicates that elite athletes, leveraging superior spatial cognitive abilities, particularly mental rotation skills (Malinowski, 2001; Schmidt et al., 2016), demonstrate enhanced proficiency in matching map features with the corresponding terrain and planning optimal routes within complex environments (Y. Liu, 2019). Mental rotation, the ability to mentally imagine the rotation of oneself or an object, represents a crucial capacity for transforming spatial representations and serves as a key indicator of spatial ability (Peng et al., 2005). It directly influences map navigation effectiveness. The map recognition process is susceptible to disruption by map rotation operations. This interference fundamentally arises because individuals must perform mental rotation to align the map perspective with the actual ground view for accurate matching of symbols with terrain features (Yan et al., 2022). Research indicates that map rotation significantly impairs map recognition efficiency in orienteering athletes, with elite athletes demonstrating superior efficiency compared to novices (Zhao et al., 2023; W. Long et al., 2025). Although elite athletes exhibit behavioral advantages in such tasks (J. Liu et al., 2024), existing research has primarily focused on maps featuring simple landmark symbols. There remains a notable lack of attention to the complex spatial structures of mountainous terrain, and particularly, a dearth of cross-modal evidence concerning the visuo-neural collaborative mechanisms under rotated conditions. Therefore, this study will investigate the differences in cognitive processing characteristics across varying map orientations. It aims to uncover the dynamic neural encoding mechanisms underlying individuals’ terrain symbol representation and provide a theoretical foundation for real-time navigation decision-making in mountainous environments.

Eye-tracking technology provides a crucial approach for analyzing map scene recognition processing. Research has found that in urban settings, when map orientation is inconsistent with the direction of travel, athletes demonstrate reduced fixation frequency and increased saccade count (Limin et al., 2023), indicating that map rotation significantly alters their visual search behavior patterns. Therefore, a key question for this study is: Within the more challenging context of mountainous terrain maps, what effects will different map orientations have on the visual search behavior of orienteering athletes during terrain representation? This constitutes the key question to be addressed in this study. While eye-tracking technology can effectively reveal the attention allocation strategies employed by orienteering athletes during map cognition (Stangl et al., 2023), eye-tracking studies alone can only describe the characteristics of visual attention distribution. They cannot reveal the underlying neural encoding mechanisms of terrain symbol processing and also lack the capacity for the systematic elucidation of visuo-neural collaborative mechanisms. According to information processing theory, the processing of visual information begins with selective attention capturing and filtering key environmental information. This information is then encoded and transmitted to the central nervous system, where the brain matches and compares immediate input with motor schemas stored in long-term memory to identify the optimal response plan. Finally, the central system issues commands to effectors, which execute corresponding actions or responses through coordination with the neuromuscular system (Schultz & Gava, 2019; Schyns et al., 2009). Therefore, it is necessary to further explore the visuo-neural collaborative mechanisms involved in orienteering athletes’ spatial representation processes under different map orientations.

Neuroimaging techniques have provided valuable insights into the study of individual spatial navigation (Patai & Spiers, 2021; Spiers & Barry, 2015), wayfinding, and spatial cognition (Epstein et al., 2017). For instance, research by Spiers et al. using functional magnetic resonance imaging (fMRI) on taxi drivers revealed activity changes in specific brain regions during navigation (Spiers & Maguire, 2007). Functional near-infrared spectroscopy (fNIRS), as a viable alternative to fMRI (Fishburn et al., 2014), offers advantages including convenience, high tolerance to motion artifacts, and high temporal resolution. It can capture cerebral hemodynamic responses during dynamic tasks in real-time, making it an ideal tool for investigating the neural mechanisms underlying orienteering athletes’ representation of map symbols in relation to real-world terrain. Research indicates that mental rotation processes activate multiple brain regions, including the prefrontal cortex, posterior parietal cortex, inferior temporal cortex, precentral cortex, among others (Schöning et al., 2007; Zacks, 2008). However, traditional mental rotation tasks have limited ecological validity. Recent studies have found that map rotation can induce activity changes in the dorsolateral prefrontal cortex and ventrolateral prefrontal cortex (Zhao et al., 2023; W. Long et al., 2025). Furthermore, the ventrolateral prefrontal cortex has been demonstrated to play a significant role in spatial navigation decision-making during wayfinding tasks (Carrieri et al., 2018). These findings consistently suggest that the prefrontal cortex plays a key role in decision-making and strategy switching during route planning and spatial navigation processes (Spiers, 2008). However, previous research has predominantly focused on the prefrontal regions and has not yet systematically investigated neural activation patterns across the whole brain. Given that the recognition and representation of map symbols involve complex processes such as information search, visual processing, and selective attention (Yesiltepe et al., 2021), they inevitably require collaborative engagement across multiple brain regions. This collaboration is achieved through neural signal transmission and synchronization, manifesting as specific brain activation patterns and functional connectivity (FC) characteristics (Tomasino & Gremese, 2016). Direct evidence demonstrates that mental rotation significantly enhances inter-regional FC strength among relevant brain areas (H. Long et al., 2021), providing compelling proof that complex spatial cognition relies on dynamic and efficient neural cooperation across multiple brain regions. Therefore, to address the aforementioned research gap and comprehensively elucidate the whole-brain collaborative mechanisms underlying orienteering athletes’ cognitive processing during terrain symbol representation tasks across varying orientations, this study employs whole-brain fNIRS. This technique will monitor key regions: dorsolateral prefrontal cortex (DLPFC), frontal pole area (FPC), orbitofrontal cortex (OFC), temporal lobe (TL), parietal lobe (PL), and visual cortex (VC). By analyzing cortical activation patterns and functional connectivity (FC) profiles under different map orientation conditions, this research aims to uncover the whole-brain collaborative neural mechanisms governing athletes’ cognitive processing characteristics in such tasks.

In summary, this study aims to investigate the effects of differently oriented terrain symbols on behavioral performance, visual attention, and neural mechanisms during terrain representation tasks in orienteering athletes. Previous research utilizing combined eye-tracking and fNIRS measurements has provided foundational evidence for interpreting the relationships between visual processing, cognition, and behavior in athletes’ terrain representation (Yu et al., 2025; Wen et al., 2024). Building on this foundation, the present study employs synchronized eye-tracking and fNIRS technology to systematically analyze behavioral performance, eye movement characteristics, and brain activity changes in orienteering athletes during terrain symbol representation tasks across varying orientations. The research hypothesis posits that orienteering athletes will exhibit significant differences in behavioral performance metrics, visual attention patterns, and neural activation characteristics across differently oriented terrain symbol representation tasks.

## 2. Materials and Methods

### 2.1. Participant

Sample size estimation was performed using G*Power v3.1 software (Faul et al., 2009). With an anticipated effect size (f) of 0.5, α level set at 0.05, and statistical power (1 − β) of 0.95 (Guo et al., 2024), a paired-sample *t*-test analysis indicated a minimum required sample size of 54 participants. Accounting for potential data invalidity or attrition, 67 orienteering athletes were ultimately enrolled.

Inclusion criteria comprised normal or corrected-to-normal vision; proficiency in fundamental orienteering knowledge; abstinence from alcohol, caffeine, medication, sleep deprivation, or insomnia within 24 h prior to testing; no history of neurological/psychiatric disorders, traumatic brain injury, or cardiopulmonary diseases; and no prior participation in similar experiments, with all participants providing written informed consent after approval of the study protocol by a university’s Academic and Ethics Committee (demographic details in Table 1).

### 2.2. Experimental Materials

**Figure 1:**
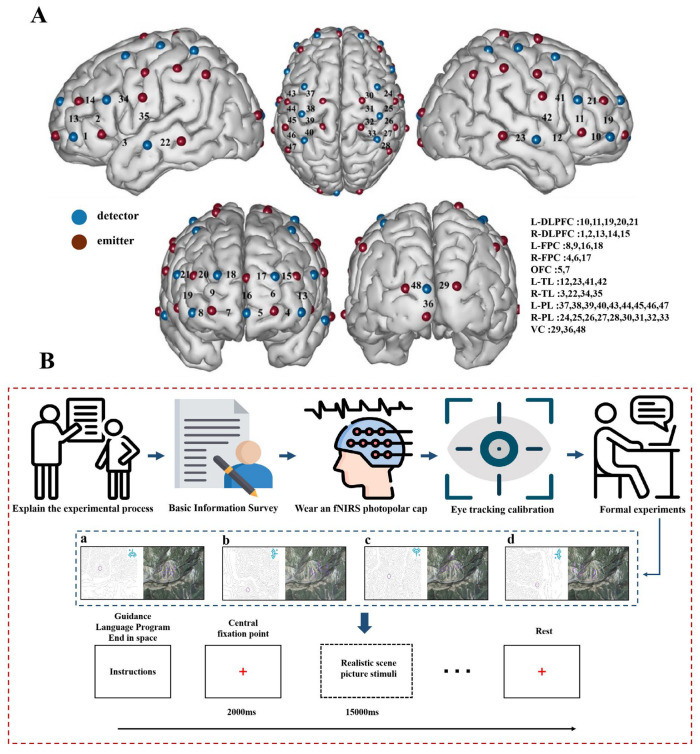
Probe placement schematic and experimental procedure flowchart. Note: (**A**): Layout of measurement channels and corresponding brain regions. (**B**): Detailed experimental workflow and stimuli. The four conditions of map orientation are presented: a, normal orientation; b, 90° rotation; c, 180° rotation; d, 270° rotation.

Experimental stimuli comprised terrain symbols in normal orientation and rotated orientations (90°, 180°, 270°). Each stimulus consisted of a 2D contour map paired with its corresponding 3D terrain view. All maps were developed by three orienteering experts. Through preliminary screening, 12 formal test symbols (6 normal orientation; 2 per rotated angle) and 4 practice symbols were selected from 48 candidate terrain symbols. See Figure 1B.

### 2.3. Experimental Apparatus

#### 2.3.1. Eye-Tracking Equipment

Eye movement data were collected using the aSee Pro desktop-mounted eye tracker (7Invensun, Beijing, China). The device employs a pupil-corneal reflection tracking technique with a sampling frequency of 120–240 Hz, 110° field of view, 0.5° tracking accuracy, and 850 nm infrared illumination. A chin rest was utilized to minimize head motion artifacts. Prior to stimulus presentation, a 9-point calibration procedure was performed, with participants maintaining a viewing distance of 70 ± 10 cm from the monitor.

#### 2.3.2. fNIRS Equipment

Hemodynamic responses in the cerebral cortex during task performance were monitored using the NirSmart-6000A (Huichuang, Danyang, China) functional near-infrared spectroscopy system. Optodes were positioned according to the 10–20 international system, covering the bilateral prefrontal cortices, temporal lobes, frontoparietal sensorimotor areas, and occipital lobe. The probe configuration comprised 24 sources + 16 detectors, forming 48 measurement channels with a sampling frequency of 11 Hz. Probe holders were secured via an elastic head cap, with hair parted to ensure optode-scalp contact. Ten regions of interest (ROIs) were defined using the LPBA40 (LONI Probabilistic Brain Atlas) anatomical atlas. See Figure 1A.

#### 2.3.3. Synchronization Protocol

fNIRS and eye-tracking data were collected simultaneously via local area network (LAN) synchronization, with Computer A handling stimulus presentation and eye movement recording while Computer B acquired fNIRS data, achieving synchronization through the eye tracker’s transmission of event markers to the fNIRS system at both stimulus onset and participant response, while thermal insulation tape secured fNIRS sensors to maintain skin contact and physical separation of the infrared camera from fNIRS probes minimized interference.

### 2.4. Experimental Design and Procedure

A single-factor between-subjects design (map orientation: normal vs. rotated) was implemented, with map orientation as the independent variable and dependent variables including behavioral measures (accuracy, reaction time), oculometric indices (saccade amplitude, pupil diameter), and fNIRS parameters (HbO_2_β values, functional connectivity strength).

This experiment was conducted in a laboratory setting. The experiment comprised practice and formal phases, incorporating two terrain symbol representation tasks (normal and rotated orientations) with identical testing procedures. Following fNIRS device specifications, participants maintained a 15 s resting state prior to task initiation to stabilize hemodynamic baselines. The formal procedure was as follows: After on-screen prompts appeared, participants pressed the spacebar to start a trial, subsequently fixated on a central “+” for 2 s, after which a 2D contour map and its corresponding 3D terrain view were presented. Participants were required to identify which labeled option (1, 2, or 3) in the 3D view corresponded to the circled location on the 2D map, responding within 15 s via designated keys (A/S/D). Each task included two practice trials and three experimental blocks (2 trials per block), with 15 s rest intervals between blocks. Total experiment duration approximated 5 min (Figure 1B).

### 2.5. Data Processing and Analysis

Behavioral metrics comprised reaction time and accuracy rate. For each participant, the mean reaction time and accuracy rate across trials during the terrain symbol representation task were calculated as primary behavioral outcomes.

Areas of Interest (AOIs) encompassing map symbols and corresponding 3D terrain renderings within the stimulus material were defined for eye-tracking analysis, with key metrics comprising saccade amplitude, pupil diameter, and spatial fixation distribution visualized as heatmaps. Data processing was conducted using aSee Studio (7Invensun, Beijing, China), implementing exclusion criteria that discarded eye-tracking data points exhibiting tracking confidence below 90% prior to statistical analysis.

fNIRS data preprocessing was performed using NirSpark v1.8.8 (Huichuang, Danyang, China), beginning with the exclusion of unqualified data in the Quality Control module via the Coefficient of Variation (CV) method and the removal of redundant segments after selecting the target experimental time period. Subsequent preprocessing steps within the Preprocess module included motion artifact correction using spline interpolation per channel, application of a 0.01–0.20 Hz band-pass filter to eliminate task-unrelated low-frequency drift and high-frequency neuro-physiological noise, and conversion of the filtered optical density data to oxygenated hemoglobin (HbO_2_) concentration changes based on the modified Beer-Lambert law. Brain activation analysis was then conducted using the GLM module, where task conditions were defined with the hemodynamic response function time-locked to task onset (spanning −15 s to 30 s, with −15 to 0 s retained as baseline and 0–30 s reflecting the single-block duration) to generate channel-wise HbO_2_ β-values; activated brain regions during tasks were identified via one-sample *t*-tests, while differences in brain activation between different map orientation tasks across channels were evaluated using paired-sample *t*-tests. Functional connectivity analysis was subsequently performed in the Network module by extracting time-series HbO_2_ concentrations per time point across brain regions, calculating Pearson correlation coefficients (r) between HbO_2_ time series of channel/region pairs, and applying Fisher-Z transformation to quantify functional connectivity strength; group-level analyses examined differences in functional connectivity strength both between channels and between brain regions for each task, with all statistical test *p*-values corrected using the false discovery rate (FDR) method.

Processed behavioral, eye-tracking, and fNIRS data were analyzed using SPSS Statistics v27.0 (IBM Corp., Armonk, NY, USA). Outliers were first identified via Tukey’s Fence method. The Shapiro–Wilk test indicated that parametric assumptions were not violated (*p* > 0.05), justifying the use of parametric tests. Paired-sample *t*-tests were employed to examine differences in metrics across terrain symbol representation tasks. Pearson correlation coefficients were computed between behavioral performance, eye-tracking metrics, and fNIRS data.

## 3. Results

### 3.1. Behavioral Performance

**Figure 2:**
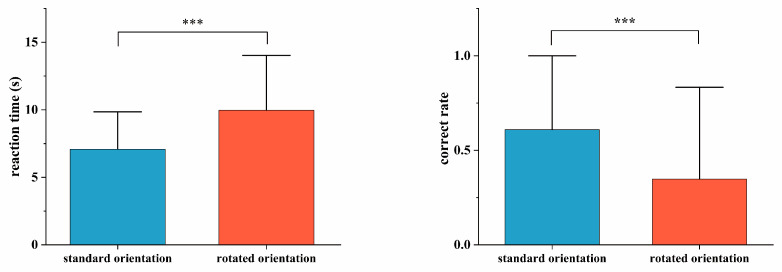
Behavioral differences in terrain symbol representation tasks across map orientations. Note: *** *p* < 0.001.

Paired-samples *t*-tests revealed significantly higher accuracy under normal map orientation compared to rotated orientation [*t* = 8.612, *p* < 0.001, Cohen’s *d* = 0.248], alongside significantly shorter reaction times [*t* = −11.069, *p* < 0.001, Cohen’s *d* = 2.131]. See Figure 2.

### 3.2. Eye Movement Results

#### 3.2.1. Saccade Amplitude and Pupil Diameter

**Figure 3:**
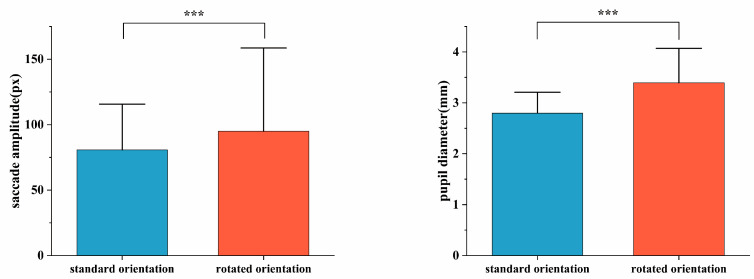
Differences in saccade amplitude and pupil diameter during terrain symbol representation tasks across map orientations. Note: *** *p* < 0.001.

Paired-samples *t*-tests indicated significantly smaller saccade amplitudes under normal map orientation compared to rotated orientation [*t* = −3.750, *p* < 0.001, Cohen’s *d* = 31.289], alongside significantly reduced pupil diameter [*t* = −13.574, *p* < 0.001, Cohen’s *d* = 0.254]. See Figure 3.

#### 3.2.2. Fixation Hotspot Map

**Figure 4:**
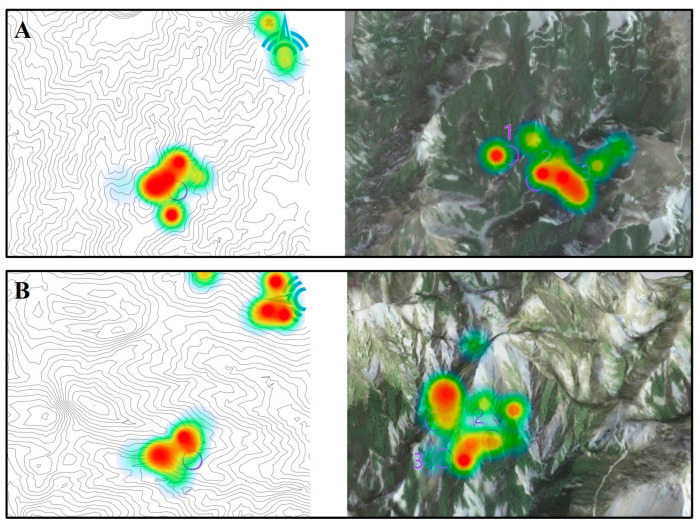
Fixation hotspot differences during terrain symbol representation tasks across map orientations. Note: (**A**) Normal orientation (**B**) Rotated orientation.

Fixation hotspots reflect athletes’ attentional distribution across visual targets, with red indicating high visual attention, yellow moderate attention, and green low attention. Analysis revealed that during both tasks, orienteering athletes demonstrated highly focused attention on key answer options. Compared with normal orientation, the rotated condition elicited significantly greater attention toward the compass. See Figure 4.

### 3.3. fNIRS Results

#### 3.3.1. Brain Activation Analysis Across Map Orientations

**Figure 5:**
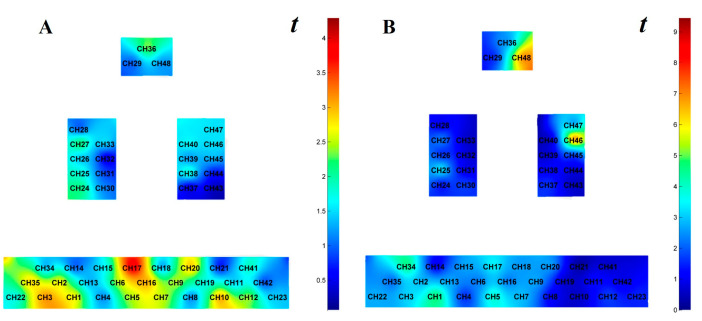
*t*-value activation maps of HbO_2_β values during terrain symbol representation tasks across map orientations. Note: (**A**): Normal orientation; (**B**): Rotated orientation.

One-sample *t*-tests were conducted on cerebral activation channels during terrain symbol representation tasks, testing against a null hypothesis value of 0 (Bai et al., 2016), revealing significantly activated channels including CH1, CH2, CH3, CH6, CH7, CH9, CH10, CH12, CH16, CH17, CH20, CH27, CH35, and CH36 under normal orientation, whereas rotated orientation showed activation in CH1, CH2, CH3, CH7, CH9, CH13, CH15, CH17, CH18, CH20, CH22, CH25, CH27, CH29, CH34, CH46, and CH48, as shown in Figure 5.

#### 3.3.2. Brain Activation Differences Across Map Orientations

**Figure 6:**
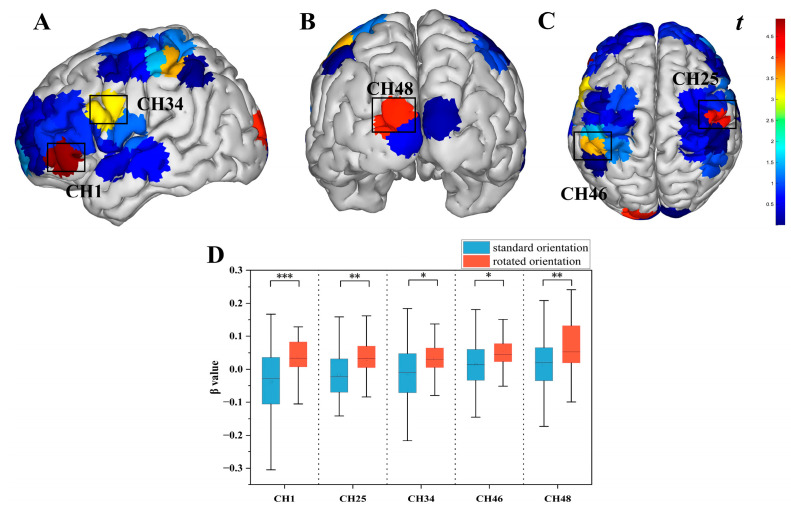
Differential *t*-value activation maps of HbO_2_β values during terrain symbol representation tasks across map orientations. Note: (**A**–**C**): Brain activation maps showing differences across orientations; (**D**): Box plot of HbO_2_β values for channels across orientations. * *p* < 0.05, ** *p* < 0.01, *** *p* < 0.001.

Paired-samples *t*-test results demonstrated significantly lower activation levels under normal orientation compared to rotated orientation across multiple channels: CH1 [*t* = −4.924, *p* < 0.001, *P*_FDR_ < 0.001, Cohen’s *d* = 0.086]; CH25 [*t* = −4.162, *p* < 0.001, *P*_FDR_ = 0.001, Cohen’s *d* = 0.063]; CH34 [*t* = −3.173, *p* = 0.002, *P*_FDR_ = 0.019, Cohen’s *d* = 0.077]; CH46 [*t* = −3.383, *p* < 0.001, *P_FDR_* = 0.012, Cohen’s *d* = 0.058]; and CH48 [*t* = −4.109, *p* < 0.001, *P*_FDR_ = 0.001, Cohen’s *d* = 0.080]. This indicates significantly reduced activation in R-DLPFC, L-PL, R-PL, R-TL, and VC during normal orientation. See Figure 6.

#### 3.3.3. Functional Connectivity Analysis Across Map Orientations

**Figure 7:**
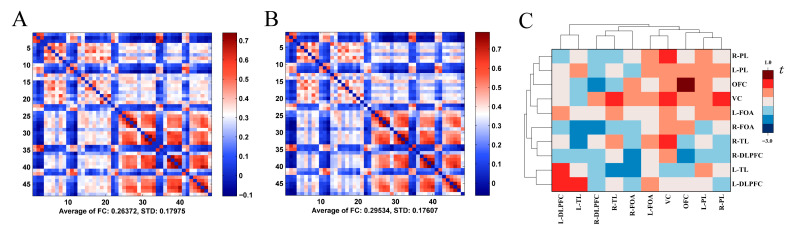
Functional connectivity visualizations during terrain symbol representation tasks across map orientations. Note: (**A**): Chord diagram of FC strength (normal orientation); (**B**): Chord diagram of FC strength (rotated orientation); (**C**): Circular clustered heatmap of t-values for FC strength differences across brain regions.

Functional connectivity results indicated an FC strength of 0.264 for the normal orientation and 0.295 for the rotated orientation. Paired-samples *t*-test results demonstrated significantly greater overall functional connectivity strength in the rotated orientation compared to the normal orientation [*t* = 32.780, *p* < 0.001, Cohen’s *d* = 0.415], as shown in Figure 7A,B. Region-based analysis revealed no statistically significant differences in FC strength for either inter-regional or homologous connections between tasks (*p* > 0.05), illustrated in Figure 7C.

### 3.4. Correlation Analysis Between Behavioral and Eye-Tracking Metrics

To investigate relationships between behavioral performance and oculomotor characteristics across map orientations, Pearson correlation analyses were conducted between task accuracy and eye-tracking metrics (saccade amplitude, pupil diameter) within each condition. Results demonstrated significant positive correlations across both conditions: under normal orientation, task accuracy correlated positively with saccade amplitude (*r* = 0.520, *p* < 0.001) and pupil diameter (*r* = 0.443, *p* < 0.001); while during rotated orientation, accuracy similarly showed positive associations with saccade amplitude (*r* = 0.298, *p* = 0.018) and pupil diameter (*r* = 0.359, *p* = 0.003). See Table 2.

### 3.5. Correlation Analysis Between Behavioral Performance and fNIRS Data

To investigate the relationship between behavioral performance and cortical activation in orienteering athletes across map orientations, Pearson correlation analyses were conducted separately for each orientation between behavioral metrics and cerebral HbO_2_β values. Since significant brain activation differences between map orientations were only observed in the R-DLPFC (Channel CH1), L-PL (Channel CH46), R-PL (Channel CH25), R-TL (Channel CH34), and VC (Channel CH48), all subsequent analyses focused exclusively on these five regions. Results indicated: under rotated orientation, no statistically significant correlations were found between behavioral performance and activated brain regions (*p* > 0.05); under standard orientation, accuracy showed a positive correlation with R-DLPFC activation (*r* = 0.372, *p* = 0.002, *P_FDR_* = 0.010). See Table 3.

### 3.6. Correlation Analysis Between Eye-Tracking Metrics and fNIRS Data

To examine relationships between oculomotor characteristics and cortical activation across map orientations, Pearson correlation analyses were conducted between eye-tracking metrics (pupil diameter, saccade amplitude) and HbO_2_β values for each orientation. Results revealed significant positive correlations under normal orientation: saccade amplitude with R-DLPFC (*r* = 0.568, *p* < 0.001, *P_FDR_* < 0.001), and R-PL (*r* = 0.363, *p* = 0.004, *P_FDR_* = 0.010), and pupil diameter with R-DLPFC (*r* = 0.383, *p* = 0.002, *P_FDR_* = 0.010); under rotated orientation, pupil diameter showed positive correlations with L-PL (r = 0.322, *p* = 0.012, *P_FDR_* = 0.030) and R-PL (r = 0.393, *p* = 0.002, *P_FDR_* = 0.010). See Table 4.

## 4. Discussion

Behavioral results indicate that map orientation significantly impacts the performance of orienteering athletes in terrain symbol representation. Specifically, under rotated map orientations, accuracy significantly decreased and reaction times significantly increased. The between-group differences supported the initial hypothesis, and the effect sizes were consistent with those reported in similar studies, falling within the medium-to-large range (Zhao et al., 2023). During map representation, orienteering athletes need to process and analyze incoming map information and match it with the real-world scene to make judgments. According to cognitive resource theory, a normal map orientation does not negatively affect their cognitive efficiency. However, when the map orientation is rotated, athletes must consume additional cognitive resources to perform mental rotation operations. This leads to prolonged cognitive processing time and reduced decision-making efficiency (Pannebakker et al., 2011; Smillie et al., 2016) This study found that reaction times increased under rotated orientations, indicating that when athletes perform mental rotation, their cognitive system requires more time to rotate the stimulus image to match the original image (Habacha et al., 2018). The decrease in accuracy indicates that, constrained by the limited nature of cognitive resources (Burge et al., 2013), the mental rotation process interferes with the precise identification of contour features, thereby reducing overall decision quality. Therefore, the rotation of map orientation affects the allocation of athletes’ cognitive resources, leading them to invest more attentional resources to complete the task.

Visual search is crucial for athletes to acquire critical information and make sound decisions in complex, dynamic competition scenarios. Eye movement results indicate that during rotated orientation tasks, orienteering athletes exhibited visual search characteristics marked by significantly increased saccade amplitude and pupil diameter. The effect sizes for these changes reached medium-to-large magnitudes, indicating that map rotation not only alters the geometric relationships within the search space but also triggers additional reconfiguration of cognitive resources (Brunye & Gardony, 2017). Existing research indicates that increased saccade amplitude reflects athletes engaging in more extensive visual scanning for in-depth map processing (Cardona & Quevedo, 2014). Meanwhile, pupil diameter—a sensitive indicator of cognitive load (Klingner et al., 2011; Laeng et al., 2011)—showed significant dilation, suggesting greater mobilization of mental resources during rotated orientation tasks (Asadi et al., 2022). According to attentional resource theory, when map orientation rotates, athletes must reconfigure their limited attentional resources to accommodate the transformation of spatial reference frames (Randall et al., 2014). Thus, increased saccade amplitude and pupil dilation serve not only as markers of effort expenditure but also as real-time signals of resource reconfiguration. Furthermore, across both task conditions, accuracy was positively correlated with both saccade amplitude and pupil diameter. This demonstrates that the heightened attentional investment and optimized visual search strategies employed by athletes during rotated tasks provide robust evidence that complex spatial cognitive tasks rely on the tight coupling between effective mobilization of cognitive resources and efficient visual information acquisition strategies (Young & Stanton, 2002).

To investigate the neural mechanisms of terrain symbol representation in orienteering athletes, this study employed whole-brain fNIRS. Results revealed significant activation across multiple brain regions during both orientation tasks, including L-DLPFC, R-DLPFC, L-FPC, R-FPC, OFC, L-TL, R-TL, L-PL, R-PL, and VC. Research indicates that mental rotation engages extensive neural networks: object recognition activates the ventral occipitotemporal pathway (“what” system), while spatial processing recruits the dorsal occipitoparietal pathway (“where system) (Koshino et al., 2005), demonstrating synergistic collaboration among regions during map orientation processing. Visual-spatial memory, fundamental to mental rotation (Ebert et al., 2024), is supported by bilateral DLPFC activation reflecting working memory and executive control functions, enabling athletes to maintain and compare spatial representations pre-post-rotation (Mutlu et al., 2020; Shelton & Pippitt, 2006). FPC activation relates to attentional resource shifting and allocation, with significant recruitment during stimulus changes in cueing paradigms (Pollmann & Manginelli, 2009). The PL serves as the core hub for spatial computation during mental rotation (Podzebenko et al., 2002), primarily calculating spatial coordinates during map rotation. Notably, right-lateralized activation emerged with significant R-TL engagement but non-significant L-TL involvement, supporting right-hemisphere dominance for spatial tasks (Barr, 1997; Hepworth & Smith, 2002; Pillon et al., 1999). OFC activation suggests reward-based evaluation of orientation choices and feature extraction during spatial decision-making (Rolls et al., 2023), while VC engagement reflects basic visual processing for map feature extraction and preliminary orientation analysis (Pollen, 2008; Silson et al., 2018). Collectively, spatial cognition relies on coordinated interactions among these regions. These findings not only validate the dual-pathway theory—where dorsal and ventral attention networks dynamically integrate goal-directed and stimulus-driven processes (Ibrayev et al., 2024; Smigasiewicz et al., 2019; Vossel et al., 2014)—but also elucidate the collaborative neural mechanisms underlying athletes’ efficient spatial information processing.

Brain activation differences revealed significantly lower activation in R-DLPFC, L-PL, R-PL, R-TL, and VC during normal orientation tasks compared to rotated orientation tasks. Notably, while these differences reached statistical significance, effect size analysis indicated that the effect sizes were generally small. The activation differences between tasks reveal a nuanced pattern of dynamic cognitive resource allocation. Specifically, the lower activation in the DLPFC during normal orientation reflects the advantage of automated processing and a relative reduction in cognitive control demands in this task, aligning with the core tenet of the neural efficiency hypothesis (Bilalic, 2018), which posits that efficient cognitive processing is often accompanied by optimized activation in specific brain regions. Conversely, the increased activation in the PL and R-TL during the rotated task corresponds to a significantly higher load for spatial mental operations and higher-order visual feature recognition (Hiew et al., 2023), requiring the recruitment of additional resources for mental simulation (Shelton & Pippitt, 2006). This task-specific activation pattern suggests that orienteering training enhances spatial representation efficiency by optimizing resource allocation within the dorsal pathway, reflecting the plasticity of spatial skills (Fernandez-Mendez et al., 2018; Ileri et al., 2023; Yang et al., 2020). Meanwhile, the study found significantly stronger global functional connectivity strength during the rotated orientation compared to the normal orientation, with effect sizes at a moderate level. This aligns with findings by (Shirzadi et al., 2025), indicating that this enhanced connectivity pattern optimizes information transfer efficiency during spatial cognitive transformation and mental rotation simulation (Cortes et al., 2023; Ecker et al., 2008). It helps mitigate the cognitive load induced by increased task complexity and further reveals a substantial elevation in the demand for inter-regional collaborative processing. Additionally, the differential correlations between oculometric indices (saccade amplitude, pupil diameter) and distinct brain regions across the two tasks further elucidate the neural dissociation of behavioral strategies. During the normal task, the association between eye movement behavior and the R-DLPFC, and R-PL reflects the coupling of top-down goal-directed attention with localized spatial attention regions (Sani et al., 2021; Valdois et al., 2019). In contrast, the association between eye movement behavior and the PL during the rotated task indicates heightened coupling driven by increased demands for spatial working memory maintenance and spatial computational processing (Macaluso, 2019; Seo et al., 2012; Silk et al., 2010). This visuocognitive coupling mechanism suggests that orienteering athletes’ ability to process landscape symbols in different orientations is reflected in the rapid mobilization of attentional and spatial working memory resources within frontoparietal regions.

## 5. Limitations and Future Research

This study investigated the neural mechanisms underlying the spatial representation reconstruction of topographic map symbols in orienteering athletes by integrating eye-tracking and whole-brain fNIRS. The experimental stimuli consisted of two-dimensional contour maps and their corresponding three-dimensional realistic scenes. The results demonstrated that orienteering athletes achieve spatial representation reconstruction by modulating the synergistic patterns between visual behavior and brain activity to accommodate changes in map orientation. However, this study has the following limitations: (1) The main limitation of this study is the lack of a control group of non-orienteers. This constraint limits our ability to definitively attribute the observed effects to orienteering expertise, as we cannot determine whether orienteering training genuinely enhances map symbol spatial cognitive abilities. Therefore, any conclusions regarding expertise-specific mechanisms should be interpreted with caution. Future studies should incorporate well-matched control groups or longitudinal designs—possibly using Linear Mixed-Effects Models (LME)—to better isolate expertise-related effects and developmental trajectories (Gath-Morad et al., 2021). (2) Presenting stimuli on a computer screen limited ecological validity. Future research could utilize Virtual Reality (VR) technology to construct immersive outdoor environments, thereby enhancing the ecological validity of the experiments. (3) The mental rotation paradigm currently employed is known to exhibit significant gender differences (H. Long et al., 2021). Future research should incorporate multiple rotation angles to systematically examine the cognitive neural mechanisms underlying the interaction between gender factors and different rotation angles. (4) This study did not include analysis of microsaccades. Future eye-tracking studies could incorporate this metric to capture more finely the dynamic changes in attentional modulation, transient fluctuations in cognitive load, and the mechanisms of information integration preceding decision-making during spatial rotation tasks (Dalmaso et al., 2017). (5) A short pre-task baseline period (15 s) was employed in this study, which may be insufficient to establish a stable hemodynamic baseline. This increases the risk of result variability and poses a limitation on the reliability of the fNIRS data interpretation. These improvements would provide more scientifically rigorous, precise, and evidence-based support for the theoretical construction and practical application of cognitive theories in orienteering.

## 6. Conclusions

This study, employing synchronized eye-tracking and fNIRS, demonstrates that map rotation significantly impairs behavioral performance in terrain symbol representation among orienteering athletes while optimizing this process through altered visual search patterns and attentional resource allocation. As mental rotation demands and cognitive load increase, heightened activation emerges in the right dorsolateral prefrontal cortex (R-DLPFC), bilateral parietal lobes (PL), right temporal lobe (R-TL), and visual cortex (VC), accompanied by enhanced functional connectivity between regions. This indicates that interregional collaboration improves information transfer efficiency to address cognitive challenges induced by orientation changes. Significant coupling between oculometric indices and neural activity reveals a neural efficiency mechanism: the frontoparietal network dynamically supports spatial representation reconstruction through rapid mobilization of attentional and spatial working memory resources. These findings suggest that orienteering-specific training should prioritize enhancing athletes’ terrain symbol representation capabilities under rotated conditions and develop interregional collaborative efficiency to optimize cognitive performance in complex environments.

## Figures and Tables

**Table 1:** Participant Demographic Characteristics.

Indicator	Male	Female
Sample size (*N*)	36	31
Athlete level	Level 1 or above	Level 1 or above
Mean age (years)	21.94 ± 1.12	21.13 ± 0.49
Mean training history (years)	4.94 ± 0.75	5.03 ± 0.48
Training frequency (hours/week)	9.61 ± 1.44	9.77 ± 0.43

**Table 2:** Correlation results between accuracy and eye-tracking metrics during terrain symbol representation tasks across map orientations.

Group	Saccade Amplitude		Pupil Diameter	
	*r*	*p*	*r*	*p*
standard orientation	0.520 *	<0.001	0.443 *	<0.001
rotated orientation	0.298 *	0.018	0.359 *	0.003

Note: An asterisk (*) denotes a statistically significant correlation.

**Table 3:** Correlation results between behavioral metrics and fNIRS indicators during terrain symbol representation tasks across map orientations.

Group	Indicator	Correct Rate		
		*r*	*p*	*P_FDR_*
standard orientation	R-DLPFC	0.372 *	0.002	0.010
	L-PL	−0.011	0.933	0.965
	R-PL	0.279	0.029	0.073
	R-TL	−0.006	0.965	0.965
	VC	0.061	0.648	0.965
rotated orientation	R-DLPFC	−0.217	0.099	0.352
	L-PL	0.166	0.209	0.352
	R-PL	−0.165	0.211	0.352
	R-TL	−0.145	0.305	0.381
	VC	0.063	0.626	0.626

Note: An asterisk (*) denotes a statistically significant correlation.

**Table 4:** Corsrelation results between eye-tracking metrics and fNIRS indicators during terrain symbol representation tasks across map orientations.

Group	Indicator	Saccade Amplitude			Pupil Diameter		
		*r*	*p*	*P_FDR_*	*r*	*p*	*P_FDR_*
standard orientation	R-DLPFC	0.568 *	<0.001	<0.001	0.383 *	0.002	0.010
	L-PL	−0.036	0.781	0.781	−0.121	0.342	0.465
	R-PL	0.363 *	0.004	0.010	0.230	0.074	0.185
	R-TL	0.117	0.373	0.466	0.115	0.372	0.465
	VC	0.274	0.037	0.061	0.094	0.479	0.479
rotated orientation	R-DLPFC	0.276	0.038	0.190	−0.038	0.770	0.936
	L-PL	0.103	0.445	0.686	0.322 *	0.012	0.030
	R-PL	0.049	0.720	0.720	0.393 *	0.002	0.010
	R-TL	−0.142	0.324	0.686	0.011	0.936	0.936
	VC	0.080	0.549	0.686	0.050	0.701	0.936

Note: An asterisk (*) denotes a statistically significant correlation.

## Data Availability

The raw data supporting the conclusions of this article will be made available by the authors on request.

## Abbreviations

The following abbreviations are used in this manuscript:

L-DLPFC	Left Dorsolateral Prefrontal Cortex
R-DLPFC	Right Dorsolateral Prefrontal Cortex
L-FPC	Left Frontal Pole Area
R-FPC	Right Frontal Pole Area
OFC	Orbitofrontal Cortex
L-TL	Left Temporal Lobe
R-TL	Right Temporal Lobe
L-PL	Left Parietal Lobe
R-PL	Right Parietal Lobe
VC	Visual Cortex
FC	Functional Connectivity
fNIRS	Functional Near-Infrared Spectroscopy
HbO_2_	Oxyhemoglobin
